# Mori Folium and Mori Fructus Mixture Attenuates High-Fat Diet-Induced Cognitive Deficits in Mice

**DOI:** 10.1155/2015/379418

**Published:** 2015-04-07

**Authors:** Hyo Geun Kim, Hyun Uk Jeong, Gunhyuk Park, Hocheol Kim, Yunsook Lim, Myung Sook Oh

**Affiliations:** ^1^Department of Oriental Pharmaceutical Science, College of Pharmacy, Kyung Hee University, 26 Kyungheedae-ro, Dongdaemun-gu, Seoul 130-701, Republic of Korea; ^2^Department of Life and Nanopharmaceutical Science, College of Pharmacy, Kyung Hee University, 26 Kyungheedae-ro, Dongdaemun-gu, Seoul 130-701, Republic of Korea; ^3^Department of Herbal Pharmacology, College of Korean Medicine, Kyung Hee University, 26 Kyungheedae-ro, Dongdaemun-gu, Seoul 130-701, Republic of Korea; ^4^Department of Food and Nutrition, Kyung Hee University, 26 Kyungheedae-ro, Dongdaemun-gu, Seoul 130-701, Republic of Korea

## Abstract

Obesity has become a global health problem, contributing to various diseases including diabetes, hypertension, cancer, and dementia. Increasing evidence suggests that obesity can also cause neuronal damage, long-term memory loss, and cognitive impairment. The leaves and the fruits of *Morus alba* L., containing active phytochemicals, have been shown to possess antiobesity and hypolipidemic properties. Thus, in the present study, we assessed their effects on cognitive functioning in mice fed a high-fat diet by performing immunohistochemistry, using antibodies against c-Fos, synaptophysin, and postsynaptic density protein 95 and a behavioral test. C57BL/6 mice fed a high-fat diet for 21 weeks exhibited increased body weight, but mice coadministered an optimized Mori Folium and Mori Fructus extract mixture (2 : 1; MFE) for the final 12 weeks exhibited significant body weight loss. Additionally, obese mice exhibited not only reduced neural activity, but also decreased presynaptic and postsynaptic activities, while MFE-treated mice exhibited recovery of these activities. Finally, cognitive deficits induced by the high-fat diet were recovered by cotreatment with MFE in the novel object recognition test. Our findings suggest that the antiobesity effects of MFE resulted in recovery of the cognitive deficits induced by the high-fat diet by regulation of neural and synaptic activities.

## 1. Introduction

Obesity is a medical condition with an increasing prevalence; for example, about one-third of American adults are now obese [1]. The causes of obesity are complex, but the most common is excessive dietary calories due to a high-fat diet [2]. Obesity is associated with not only several chronic diseases, including fatty liver, heart disease, type 2 diabetes mellitus, certain types of cancer, and osteoarthritis [1, 3], but also impairment of certain brain functions [4]. Although the relationship between obesity and adverse effects in the brain remains unclear, studies have suggested that obesity and body fat deposition play an important role in the pathogenesis of certain brain-related disorders [5].

Recent studies—clinical and preclinical—have suggested that obesity impairs cognition [4]. For example, people with high energy consumption are at an increased risk for Alzheimer's disease [6]. A carbohydrate-enriched high-fat diet impairs learning and memory and synaptic plasticity by affecting brain-derived neurotrophic factor and cyclic AMP-response element-binding protein and activating NADPH oxidase activity [7]. A high-fat diet also reduces neurogenesis in the hippocampus. Additionally, short-term feeding of a high-fat diet can impair attention and visual memory [4]. The exact causes of those impairments have still not been determined, but obesity induced by a high-fat diet clearly results in cognitive deficits with reduced hippocampal function.

*Morus alba* L., from the Moraceae family, is a short-lived, small-to-medium-sized mulberry tree. The species is native to northern China and is widely cultivated and naturalized elsewhere [8]. The leaves, bark, branches, and fruits of* Morus alba* L. have been used for various medicinal purposes from ancient times to the present, including treating fever, protecting the liver, improving eyesight, strengthening joints, facilitating the discharge of urine, and lowering blood pressure [9]. Particularly the leaves and fruits of* Morus alba* L. have been widely applied for medicinal uses due to their various pharmacological effects. Mori Folium, the leaves of* Morus alba* L., have antimicrobial, antioxidant, antianxiety, antihyperglycemia, hypocholesterolemic, hypotriglyceridemic, and antiobesity effects [10–14]. Mori Fructus, the fruits of* Morus alba* L., have antioxidant, anti-inflammatory, anticancer, antiobesity, antidiabetic, immunoregulatory, and hypolipidemic effects [15–19]. Additionally, previous research suggests that combined treatment with mulberry leaf and fruit is more effective in preventing obesity effects than either treatment alone; the mixture inhibited obesity-induced inflammation and oxidative stress [20, 21]. Therefore, we hypothesized that the leaves and fruits of* Morus alba* L. could be beneficial in high-fat diet-induced cognitive deficits due to their antiobesity effects. In the present study, we evaluated the effects of the leaves and fruits of* Morus alba* L. mixture on cognitive deficits induced by obesity due to a high-fat diet in mice, with a focus on neural and synaptic activities.

## 2. Materials and Methods

### 2.1. Materials

High-fat diet (D12451, 45% kcal fat) and normal diet (D12450B, 10% kcal fat) were purchased from Research Diets, Inc. (New Brunswick, NJ, USA). Rabbit polyclonal antipost synaptic density protein 95 (PSD95) and rabbit polyclonal anti-c-Fos were purchased from Abcam (Cambridge, UK). Biotinylated goat anti-mouse antibody, biotinylated goat anti-rabbit antibody, and avidin-biotin complex (ABC) were purchased from Vector Labs, Inc. (Burlingame, CA, USA). Mouse monoclonal anti-synaptophysin (SYN), paraformaldehyde (PFA), 3,3-diaminobenzidine (DAB), sodium chloride, sucrose, ethanol, and phosphate buffered saline (PBS) were purchased from Sigma-Aldrich (St. Louis, MO, USA).

### 2.2. Preparation of the Extract from Mori Folium and Mori Fructus

The Mori Folium and Mori Fructus were obtained from Yangpyeong Agricultural Development & Technology Center (Yangpyeong, Korea). The raw materials were dried using a freeze-dryer and broken into bite-size pieces (Mori Folium: 1 kg, Mori Fructus: 1 kg). Then, they were separately extracted with 70% ethanol for 24 h. The extract was filtered, evaporated, and lyophilized. The powder (yield: Mori Folium, 20%; Mori Fructus, 28%) was kept at −20°C. These functional ingredients were previously standardized using 1-deoxynojirimycin, cyaniding-3-glucoside, rutin,, and resveratrol [20]. Based on the previous report [21], the dried extracts of Mori Folium and Mori Fructus were mixed with 2 : 1 ratio, respectively, and the mixture (MFE) was dissolved in distilled water (D.W) before each experiment.

### 2.3. Animals and Drug Treatment

Male C57BL/6 mice (4 weeks, 23–25 g) were purchased from the Samtako Bio Korea (O-San, Korea). Animals were housed 3 per cage, had free access to water and food, and were maintained under constant temperature (23 ± 1°C), humidity (60 ± 10%), and a 12 h light/dark cycle. The animals were fed a normal diet (10% fat by energy) or a high-fat diet (45% fat by energy) for 9 weeks. The mice were randomly divided into 5 groups (*n* = 6 in each group): (1) a vehicle-treated and normal diet supplied group (normal group), (2) a vehicle-treated and high-fat diet supplied group, (3) a 0.2 g/kg/day MFE-treated and high-fat diet supplied group, (4) a 0.5 g/kg/day MFE-treated and high-fat diet supplied group, and (5) a 1 g/kg/day MFE-treated and high-fat diet supplied group. MFE was dissolved in D.W and administered orally once a day for the next 12 weeks. The normal group was administered with an equal volume of D.W. Animal treatment and maintenance were carried out in accordance with the Principle of Laboratory Animal Care (NIH publication number 85-23, revised 1985) and the Animal Care and Use Guidelines of Kyung Hee University, Seoul, Korea.

### 2.4. Novel Object Recognition Test

The novel object recognition test was performed according to the method described previously [22]. The experiment was carried out in a grey open field box (45 cm × 45 cm × 50 cm). Prior to the test, mice were habituated to the test box for 5 min without objects. After a habituation period, mice were placed into the test box with two identical objects and allowed to explore for 3 min. The objects used in this study were wooden blocks of the same size but different shape. The time spent by the animal exploring each object was measured (defined as the training session). Twenty-four hours after training session, mice were allowed to explore the objects for 3 min, in which familiar object used in the previous training session was placed with a novel object. The time that the animals spent exploring the novel and the familiar objects was recorded (defined as the test session). The animals were regarded to be exploring when they were facing, sniffing, or biting the object. The test box and objects were cleaned with 70% ethanol between sessions. Results were expressed as percentage of novel object recognition time (time percentage =* t*-novel/[*t*-novel +* t*-familiar] × 100).

### 2.5. Brain Tissue Preparation

7 days after behavioral test, mice were immediately anesthetized by mixture of Zoletil 50 and Rompun solution (3 : 1 ratio, 1 mL/kg,* i.m.*) and perfused transcardially with 0.05 M PBS and then fixed with cold 4% PFA in 0.1 M phosphate buffer. Brains were removed and postfixed in 0.1 M phosphate buffer containing 4% PFA overnight at 4°C and then immersed in a solution containing 30% sucrose in 0.05 M PBS for cryoprotection. Serial 30 *μ*m thick coronal sections were cut on a freezing microtome (Leica Microsystems Inc., Nussloch, Germany) and stored in cryoprotectant (25% ethylene glycol, 25% glycerol, and 0.05 M phosphate buffer) at 4°C until use for immunohistochemistry.

### 2.6. Immunohistochemistry

For immunohistochemical detection of c-Fos, PSD95, and SYN, the free floating sections were incubated with anti-c-Fos (1 : 1000 dilutions), anti-PSD95 antibody (1 : 500 dilutions), and anti-SYN (1 : 200 dilution), respectively, overnight at 4°C in the presence of 0.3% triton X-100 and 3% normal horse serum. Then, they were incubated with biotinylated anti-secondary antibodies IgG (1 : 200 dilution) for 90 min, followed by incubation in ABC (1 : 100 dilution) for 1 h at room temperature. Peroxidase activity was visualized by DAB in 0.05 M tris-buffered saline (pH 7.6). After every incubation step, they were washed three times with PBS. The sections were mounted on gelatin-coated slides, dehydrated with an ascending alcohol, cleared with xylene, and cover-slipped using histomount medium. Quantification of effect in brain tissue sections was performed by measuring the optical density of c-Fos, PSD95, and SYN immunoreactivity (IR) in the stratum lucidum (SL) of CA3 region at ×400 and ×40 magnifications using ImageJ software (Bethesda, MD, USA) and then presented as a percent of the normal group values. The images were photographed with a research microscope (BX51T-32F01; Olympus Corporation, Tokyo, Japan).

### 2.7. Statistical Analyses

All statistical analyses were conducted using the software GraphPad Prism Version 5.0 (GraphPad Software, San Diego, CA). Values are expressed as the mean ± standard error of mean (SEM). The mean ± SEM for each treatment group was compared by one-way analysis of variance, followed by the Tukey's post hoc test. Differences with a* P* value less than 0.05 were considered statistically significant.

## 3. Results

### 3.1. Effect of MFE on Body Weight Gain in Mice with High-Fat Diet

**Figure 1:**
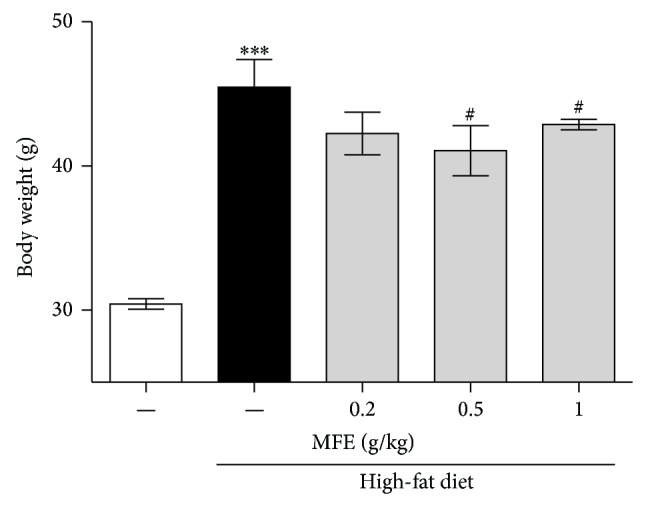
Effects of MFE on high-fat diet-induced body weight gain in male C57BL/6 mice. The mice received a normal, high-fat, or high-fat diet supplemented with MFE for 21 weeks. Values are expressed as the mean ± SEM (*n* = 6 mice per group). ^∗∗∗^ *P* < 0.001 as compared to the normal group. ^#^ *P* < 0.05 as compared to the high-fat diet group.

To examine the effect of MFE on obesity, we measured body weight of the mice to make a judgment of obesity ratio. Mice fed high-fat diet for 21 weeks showed 12.80 ± 0.85 g of weight gain versus the normal group showed 4.42 ± 0.25 g of weight gain. However, 0.2, 0.5, and 1 g/kg MFE treated mice for 12 weeks with high-fat diet showed 5.92 ± 1.2 g, 5.60 ± 0.92 g, and 8.40 ± 0.80 g of weight gain (Figure 1).

### 3.2. Effect of MFE on Neural Activity in Obesity Mouse Hippocampus

**Figure 2:**
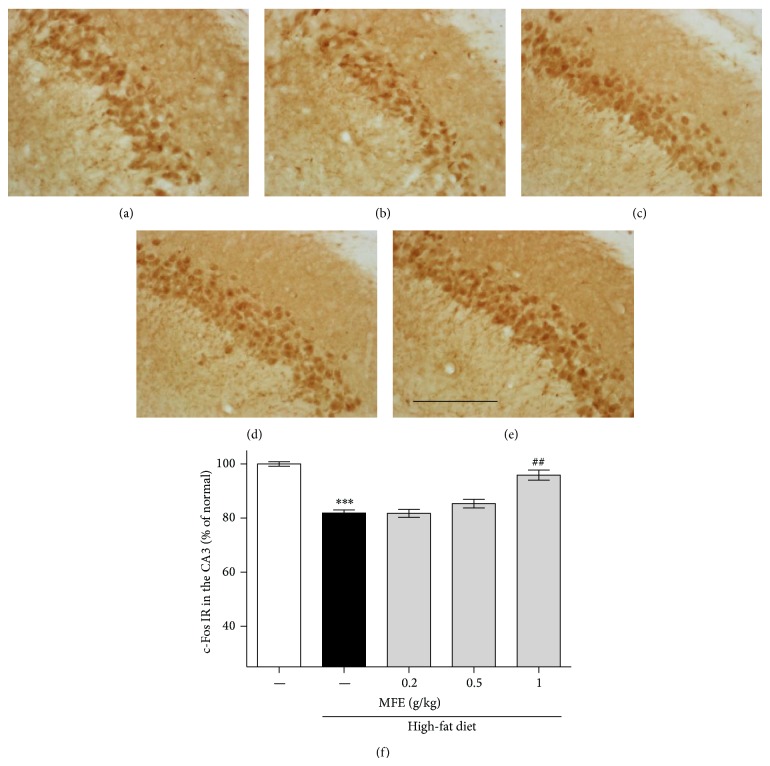
Effect of MFE on neuronal activity loss induced by high-fat diet in CA3 region of the hippocampus. The recovery effect of MFE on high-fat diet-induced dysfunction of neural activity was investigated using c-Fos immunohistochemistry (f). Representative photomicrographs are shown for the CA3 region of each group ((a)–(e)): (a) normal groups; (b) high-fat diet group; ((c), (d), and (e)) high-fat diet/MFE group (0.2, 0.5, and 1 g/kg/day treated, resp.). Scale bar = 200 *μ*m. Values are expressed as the mean ± SEM (*n* = 6 mice per group). ^∗∗∗^ *P* < 0.001 as compared to the normal group. ^##^ *P* < 0.01 as compared to the high-fat diet group.

To investigate the effects of MFE on the neuronal firing in the SL of CA3 region of the hippocampus, we performed immunostaining using c-Fos antibody in the high-fat diet-induced obesity mice. High-fat diet-induced obesity mice showed less immunoreactivity of c-Fos in the CA3 than that of the normal mice. However, MFE at 1 g/kg for 12 weeks treatment recovered the decrease of neural activity induced by obesity showing 95.88% of the normal group (Figure 2). Therefore, we could suggest that MFE treatment regulates action potential of neurons in the hippocampus of the obese mice.

### 3.3. Effect of MFE on Synaptic Activity in Obesity Mouse Hippocampus

**Figure 3:**
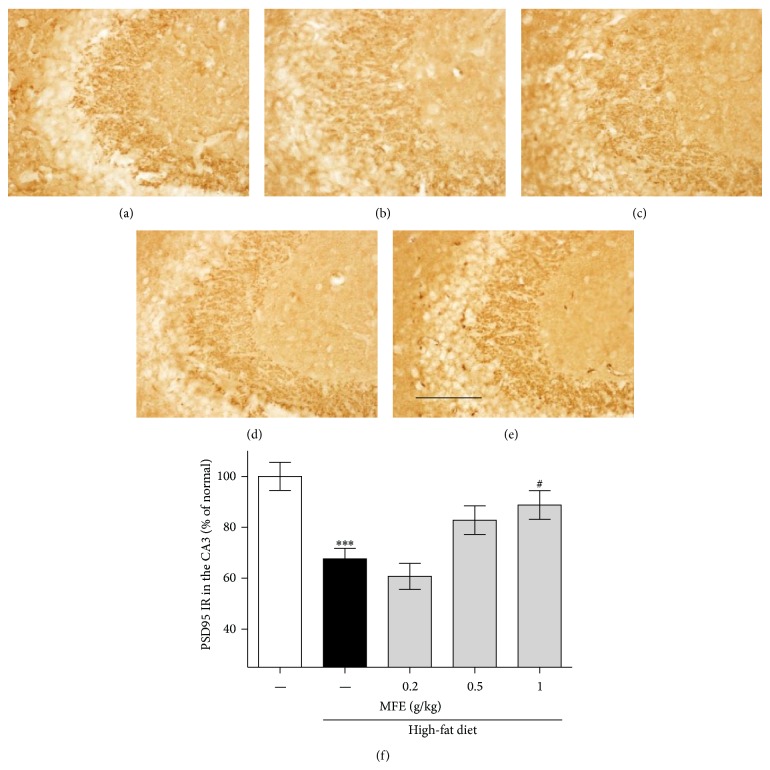
Effect of MFE on postsynaptic loss induced by high-fat diet in CA3 region of the hippocampus. The recovery effect of MFE on high-fat diet-induced synaptic dysfunction was investigated using PSD95 immunohistochemistry (f). Representative photomicrographs are shown for the CA3 region of each group ((a)–(e)): (a) normal groups, (b) high-fat diet group, and ((c), (d), and (e)) high-fat diet/MFE group (0.2, 0.5, and 1 g/kg/day treated, resp.). Scale bar = 200 *μ*m. Values are expressed as the mean ± SEM (*n* = 6 mice per group). ^∗∗∗^ *P* < 0.001 as compared to the normal group. ^#^ *P* < 0.05 as compared to the high-fat diet group.

**Figure 4:**
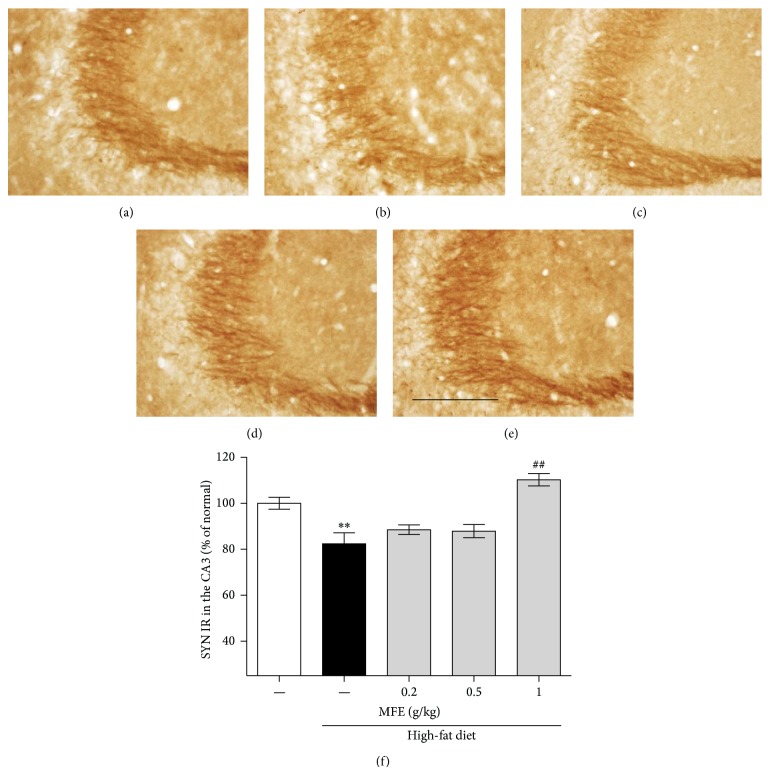
Effect of MFE on presynaptic loss induced by high-fat diet in CA3 region of the hippocampus. The recovery effect of MFE on high-fat diet-induced synaptic dysfunction was investigated using SYN immunohistochemistry (f). Representative photomicrographs are shown for the CA3 region of each group ((a)–(e)): (a) normal groups, (b) high-fat diet group, and ((c), (d), and (e)) high-fat diet/MFE group (0.2, 0.5, and 1 g/kg/day treated, resp.). Scale bar = 200 *μ*m. Values are expressed as the mean ± SEM (*n* = 6 mice per group). ^∗∗^ *P* < 0.01 as compared to the normal group. ^##^ *P* < 0.01 as compared to the high-fat diet group.

To determine the effects of MFE on synaptic activity in the SL of CA3 region of the hippocampus, we performed immunostaining using PSD95 and SYN antibodies in the high-fat diet-induced obesity mice. High-fat diet-induced obesity decreased after and before synaptic activity, showing 67.62% and 82.34% of PSD95 and SYN immunoreactivities of the normal group, respectively. However, MFE treatment for 12 weeks showed dose-dependent recovery effects, showing 88.74% and 110.19% at 1 g/kg dose (Figures 3 and 4). From these results, MFE could regulate synaptic activity in the hippocampus of the obese mice.

### 3.4. Effect of MFE on Cognitive Function in Obesity Mouse

**Figure 5:**
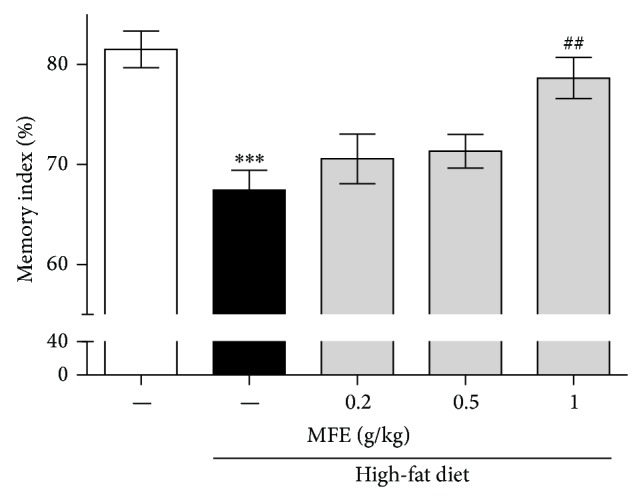
Effect of MFE on memory impairment induced by high-fat diet. Novel object recognition test was carried out at 20 weeks after high-fat diet supplied. Values are expressed as the mean ± SEM (*n* = 6 mice per group). ^∗∗∗^ *P* < 0.001 as compared to the normal group. ^##^ *P* < 0.01 as compared to the high-fat diet group.

To investigate the effect of MFC on cognitive impairment induced by obesity, we performed a novel object recognition task. The normal mice spent more time exploring the novel object than the familiar object during the test session showing 81.51% of memory index. In contrast, the high-fat diet-induced obesity mice spent less time on the novel object showing 67.45% of memory index. However, MFE treatment significantly improved obesity-induced cognitive deficits by 78.63% (Figure 5). However, no significant differences in exploratory preferences were found for any of the groups during the training session (data not shown). These results indicated that MFE recovered memory function in the obesity mice through regulation of neural action potential and synapse of neurons in the hippocampus.

## 4. Discussion

In this study, we demonstrated that the MFE restored cognitive function by recovering neural and synaptic activity in a high-fat induced obesity mouse model. Previously, we optimized the mixture of Mori Folium and Mori Fructus at a 2 : 1 ratio by measuring body weight and body fat size in high-fat diet mice [21]. In this study, we used this optimized mixture extract (MFE) and confirmed that the weight loss was dose-dependent. Generally, mice fed a high-fat diet are used to model the human diet-induced obese state [4]. This state is considered to play an important role in cognitive decline during normal aging. The mechanism of the relationship remains unclear, but common aspects of both conditions include brain insulin resistance, vascular disease, inflammation, and oxidative stress [23]. Additionally, caloric restriction and exercise, which are common methods of losing body weight, can practically slow brain aging, including cognitive decline [1, 23]. In this study, mice fed a high-fat diet for 21 weeks exhibited significant body weight gain, while mice coadministered MFE for the final 12 weeks exhibited significant body weight loss (Figure 1). Thus, we investigated whether MFE would benefit brain function in this model.

Next, we performed immunohistochemistry using anti-c-Fos, anti-PSD95, and anti-SYN antibodies to investigate whether MFE influenced hippocampal neurons. c-Fos may participate in learning and memory formation [24]; c-Fos knockout mice exhibit disruption in memory function [25]. Thus, expression of c-Fos is regarded as a marker of neural activity, in terms of firing action potentials in neurons [26]. In this study, obese mice exhibited reduced c-Fos immunoreactivity in the CA3 area of the mouse hippocampus, but MFE treatment inhibited the reduction in c-Fos expression (Figure 2). Previous research has revealed that long-term dietary restriction modulates synaptic protein expression in the hippocampus [1]. Additionally, a high-fat diet contributes to reduced brain-derived neurotrophic factor (BDNF) [27], and reduced BDNF secretion contributes to synaptic vesicle docking malfunction and synaptic protein distribution errors [28]. Thus, we examined SYN and PSD95. SYN is present in neuroendocrine cells, in which it is a presynaptic protein implicated in neurotransmitter release and synapse formation [29]. Recent research has shown that knockdown of synaptophysin creates cognitive dysfunction in mice, such as impaired object novelty recognition and reduced spatial learning [30]. PSD95 is located almost exclusively in the postsynaptic density of neurons; it is a major neuronal protein that is associated with receptors and cytoskeletal proteins and acts to maintain the synaptic structure [31]. PSD95 plays important roles in spatial learning in mice [32]. In this study, we found that MFE coadministered mice exhibited significant recovery of pre- and postsynaptic activities in the CA3 of the hippocampus, whereas high-fat diet-fed mice exhibited less synaptic activity than normal mice (Figures 3 and 4). Finally, we confirmed the effect on cognitive function by performing a behavioral test, NORT. In the retention phase, the high-fat diet group spent more time at the familiar object than the normal mice. This suggests that high-fat diet-induced obesity disrupted hippocampus-dependent memory consolidation [33]. However, the MFE cotreated group exhibited recovered recognition memory function (Figure 5). Collectively, MFE exerted beneficial effects on cognitive deficits in obese mice, apparently by regulating neural activity, presynaptic activity, and postsynaptic activity. Mori Folium and Mori Fructus contain beneficial phytoconstituents such as resveratrol, rutin, quercetin, and cyanidin-3-glucoside, all of which help prevent obesity and improve memory. These constituents of MFE may be responsible for the effects observed in this study [34–39]. However, further investigation is necessary to fully understand both which phytoconstituents are essential and MFE's neurotransmitter-releasing and molecular mechanisms of action.

## 5. Conclusions

The present study showed that MFE exerted a supportive effect on weight loss in mice with high-fat diet-induced obesity. Additionally, decreased c-Fos, SYN, and PSD95 immunoreactivity in the SL of the CA3 region of the hippocampus in mice were blocked by MFE treatment in the same model. Thus, MFE improved neural and synaptic activity. Moreover, MFE exerted an effect against obesity-induced cognitive deficits in mice fed a high-fat diet. Thus, MFE is a potential candidate agent for maintaining memory function in obesity by regulating neural and synaptic actions.
